# Medical patients’ affective well-being after emergency department admission: The role of personal and social resources and health-related variables

**DOI:** 10.1371/journal.pone.0212900

**Published:** 2019-03-20

**Authors:** Lukas Faessler, Jeannette Brodbeck, Philipp Schuetz, Sebastian Haubitz, Beat Mueller, Pasqualina Perrig-Chiello

**Affiliations:** 1 Institute of Psychology, University of Bern, Bern, Switzerland; 2 Medical University Department, Kantonsspital Aarau, Aarau, Switzerland; University of Mississippi Medical Center, UNITED STATES

## Abstract

**Background:**

Medical emergency admissions are critical life events associated with considerable stress. However, research on patients’ affective well-being after emergency department (ED) admission is scarce. This study investigated the course of affective well-being of medical patients following an ED admission and examined the role of personal and social resources and health-related variables.

**Methods:**

In this longitudinal survey with a sample of 229 patients with lower respiratory tract infections and cardiac diseases (taken between October 2013 and December 2014), positive and negative affect was measured at ED admission (T1) and at follow-up after 7 days (T2), and 30 days (T3). The role of personal and social resources (emotional stability, trait resilience, affect state, and social support) as well as health-related variables (self-rated health, multimorbidity, and psychological comorbidity) in patients’ affective well-being was examined by controlling for demographic characteristics using regression analyses.

**Results:**

The strength of the inverse correlation between positive and negative affect decreased over time. In addition to health-related variables, higher negative affect was predicted by higher psychological comorbidity over time (T1–T3). In turn, lower positive affect was predicted by lower self-rated health (T1–T2) and higher multimorbidity (T3). In terms of personal and social resources, lower negative affect was predicted by higher emotional stability (T2), whereas higher positive affect was predicted by stronger social support (T1–T2).

**Conclusion:**

Knowledge about psychosocial determinants–personal and social resources and health-related variables–of patients’ affective well-being following ED admission is essential for designing more effective routine screening and treatment.

## Introduction

An emergency department (ED) admission can be considered a stressful life event mostly associated with adverse effects on patients’ emotional state. In fact, around 20% of medical patients suffer from acute emotional distress (anxiety and worry) at ED admission [1]. Much of what we know about emotional distress in medical ED patients is related mainly to psychological symptoms such as depression and anxiety. A recent literature review showed that in general, almost half of ED patients are at risk of emotional distress, although prevalence rates varied widely across studies depending on the instruments used [2]. Furthermore, the existing literature has shown that most medical ED patients adapt quite well over time. However, about one third of these patients were identified as still at risk of emotional distress two weeks following ED admission [3]. A similar picture of adaptation was shown in longitudinal studies with other medical patient populations, which focused mainly on the presence or absence of psychological symptoms [4–7]. However, little is known to date regarding the course of patients’ affective well-being, i.e. negative and positive affect. Considering this research gap, the overall aim of the present study is to examine the 7- and 30-day affective well-being of medical patients following an ED admission.

We base our research on two complementary theoretical models. The first of these, the dynamic model of affect (DMA) [8], assumes that, in times of low stress, positive and negative affective systems function relatively independently because of maximal flexibility in information processing. In contrast, under conditions of stress, information processing becomes more simplified and rapid, leading to an increasingly inverse relationship between positive and negative emotions. The DMA has received support from various empirical studies on pain patients. For example, positive and negative affect were strongly negatively correlated on high pain days compared to low pain days [9]. With respect to the DMA, higher levels of positive or negative affect in times of stress can be crucial for coping with critical life events such as an ED admission.

Our complementary theoretical approach refers to the role of personal resources such as personality traits, which are considered protective factors for emotional adaptation to stressful life events [10]. A study showed that cardiac patients with lower levels of neuroticism experienced lower levels of emotional distress following ED admission [11]. Other researchers identified trait resilience as an important personal resource for psychological adaptation which is defined as the capacity to overcome adversity in the face of stress [12]. Higher trait resilience was associated with faster cardiovascular recovery and lower emotional distress, such as fewer depressive symptoms, following a stressful event [13,14]. Resilient patients also report fewer stress disorder symptoms during acute myocardial infarction [15]. In addition to personal resources, social support was also found to be related to better emotional adaptation. Studies have shown that lower levels of emotional distress in patients following an acute cardiac event were predicted by stronger social support [7,16].

In addition to personal and social resources, health-related variables are indeed important correlates of patients’ emotional states. A systematic literature review [2] revealed that patients’ emotional distress at ED admission was related to higher chronic medical conditions and comorbidity, poorer self-rated health state, and psychopathological conditions. In fact, lower initial psychological and physical functioning in hospitalised medical patients was associated with the persistence of emotional distress three months following discharge [17]. Furthermore, evidence from a recent study suggests that poorer adaptation in medical patients in terms of emotional distress 30 days after ED admission was predicted by the initial clinical presentation such as comorbid psychological symptoms [18].

Investigating affective well-being (instead of or in addition to psychological symptoms) should provide a finer-grained and more comprehensive understanding of affective adaptation to ED admission. Prospective epidemiological studies have suggested that affective well-being is a protective factor against mortality and morbidity in medically ill populations [19]. Thus, knowledge about the resources for affective well-being in medical patients following ED admission could be essential in identifying intervention methods that would improve well-being and overall state of health. Against this background, we explored patients’ affective well-being in terms of its determinants and in terms of positive and negative affect at ED admission and 7- and 30-day follow-up. The study concentrated on acute lower respiratory tract infections and cardiac diseases representing the predominant diagnoses in ED patients.

Based on the theoretical models described above and on the researchers’ own previous empirical findings, our research questions and expectations were as follows:

1. How are personal and social resources and health-related variables related to medical patients’ level of negative and positive affect at ED admission (T1)? We expected that patients with better personal resources (higher positive affect, trait resilience, and emotional stability), stronger social support, and better health-related variables (less multimorbidity, lower psychological comorbidity, and better self-rated health) would exhibit significantly lower levels of negative affect at ED admission (T1) compared to patients with poorer personal and social resources and health-related variables.
2. What is the role of personal and social resources and health-related variables in predicting patients’ affective well-being at ED admission, at day 7 (T2), and at day 30 (T3)? We expected that lower levels of negative affect at ED admission (T1), at day 7 (T2), and at day 30 (T3) would be best predicted by better personal resources (higher emotional stability, trait resilience, and positive affect), stronger social support, and better health-related variables (lower psychological comorbidity).

Due to the lack of prior studies, we do not have any hypotheses regarding patients’ positive affect.

## Materials and methods

### Design and procedure

This longitudinal study is part of the TRIAGE project “Optimizing Triage and Hospitalisation in Adult General Medical Emergency Patients” [20]. The present study included medical patients who were hospitalised through the ED, for acute lower respiratory tract infections and cardiac diseases, in a hospital in Northwestern Switzerland between October 2013 and December 2014 during daytime hours (9:00 a.m. to 5:00 p.m.) from Monday to Friday. The Swiss hospital (Cantonal Hospital Aarau) is a 600-bed tertiary-care hospital in which most medical admissions enter the hospital through the ED. All procedures were in accordance with the ethical standards of the responsible committee. Because it was an observational quality control study, the Institutional Review Board (IRB) approved it and waived the need for individual informed consent (IRB: Ethikkommission Kanton Aargau [EK 2012/059]). The study was registered at the ClinicalTrials.gov registration website (http://www.clinicaltrials.gov/ct2/show/NCT01768494), and the study protocol was published previously [21].

### Patient sample

The sample consisted of patients who sought ED care for medical health issues and who met our inclusion criteria. The inclusion criteria were as follows: (a) a diagnosis of acute coronary syndrome and heart failure or lower respiratory tract disease (pneumonia, chronic obstructive pulmonary disease, asthma, or bronchitis), (b) age ≥ 18 years, (c) admission to ED, and (d) a stable medical condition that allowed for an interview. The exclusion criteria were as follows: (a) an immediate need for transfer to the intensive care unit (ICU), (b) not alerted and oriented to person, place, time, and event, (c) initial treatment because of substance abuse, (d) insufficient German language skills, (e) terminal illness, or (f) unwillingness to participate.

### Data collection

Upon ED admission, all eligible patients provided a medical history and underwent a physical examination during which their diagnosis, comorbidities, and demographic characteristics were recorded. In the ED department, in the presence of a clinical psychologist, patients completed an electronic questionnaire that assessed affective well-being (negative and positive affect states) and health-related conditions (psychological symptoms and self-rated health). Throughout the hospital stay, all patients included in the study were monitored by the research team and were asked, seven days after ED admission, to complete an electronic questionnaire that assessed affective well-being and additional psychological variables (emotional stability, trait resilience, and social support). If patients were discharged before day 7, they were interviewed by telephone. A follow-up telephone questionnaire, again assessing affective well-being, was administered by the research team 30 days after ED admission. Information was entered into a case report form and stored in a centralised, password-secured databank (SecuTrial).

### Measures

#### Affective well-being

The short form of the Positive and Negative Affect Schedule [22] was used to assess patients’ affective well-being. This 10-item instrument comprises two subscales with five items each; the items ask participants to rate adjectives related to negative and positive affect. Participants rated the adjectives according to the “extent you feel this way in general” on a 5-point Likert scale (1 = very slightly or not at all to 5 = very much).

#### Personal resources

A brief version of the Resilience Scale [23,24] was used to measure trait resilience. This instrument consists of 11 items with answer options ranging from 1 = I don’t agree to 7 = I agree completely.

Emotional stability was assessed using the neuroticism short scale version of the Big Five Inventory [25]. It consists of two items scored on a scale from 1 = disagree strongly to 5 = agree strongly.

#### Social resources

Social support was measured with the Short-Form Social Support Questionnaire [26]. This questionnaire consists of seven items and is considered to be a common and efficient way to achieve a global score of perceived social support. Each item is answered on a 5-point Likert scale.

#### Health-related variables

Multimorbidity was defined by the sum of active diagnoses, each of which was represented by one point (e.g. pneumonia, hypothyroidism because of Hashimoto’s thyroiditis, and chronic renal failure = 3), and was collected from the patient’s medical history. Based on the main diagnosis, participants were categorised into those with cardiac diseases and those with lower respiratory tract infections.

Patients’ self-rated health was assessed using the EuroQol visual analogue scale (VAS) for the rating of own health state and the common core of different domains of health states [27]. The VAS was verbally administered, and the respondents had to rate their composite health state on the Numerical Rating Scale from 0 to 100 (0 = worst imaginable health state, 100 = best imaginable health state).

Psychological comorbidity was assessed using the K6, a screening instrument for psychological distress [28,29]. The K6 consists of six questions asking subjects to rate “how often they felt nervous, hopeless, restless or fidgety, so depressed that nothing could cheer them up, that everything was an effort and worthless.” The respondents rate how often the symptoms occurred within the last 30 days on a 5-point Likert scale, ranging from 1 = none of the time to 5 = all of the time.

### Statistical analyses

We computed correlations between all variables in the models. We then performed simple regression analyses for the univariate associations between independent variables (personal and social resources and health-related conditions) and dependent variables (negative and positive affect) for all three time points. Based on findings of the regression analyses, we included all significant independent variables or variables with a standardised regression coefficient of .1, which represented a weak effect in the multiple regression models, to predict negative and positive affect. The multiple regression models were controlled for age and gender. We also included affect baseline levels for predicting affect at T2 and T3. All data analyses were performed with Stata Version 12.1 (StataCorp LP, College Station, Texas, USA). The normality of residuals was tested with skewness and kurtosis tests [30]. To detect outliers, we used the BACON algorithm [31]. If residuals were found to be asymmetrically distributed, a robust regression analysis was used. The independent variables were tested for multicollinearity prior to regression analyses.

## Results

**Fig 1:**
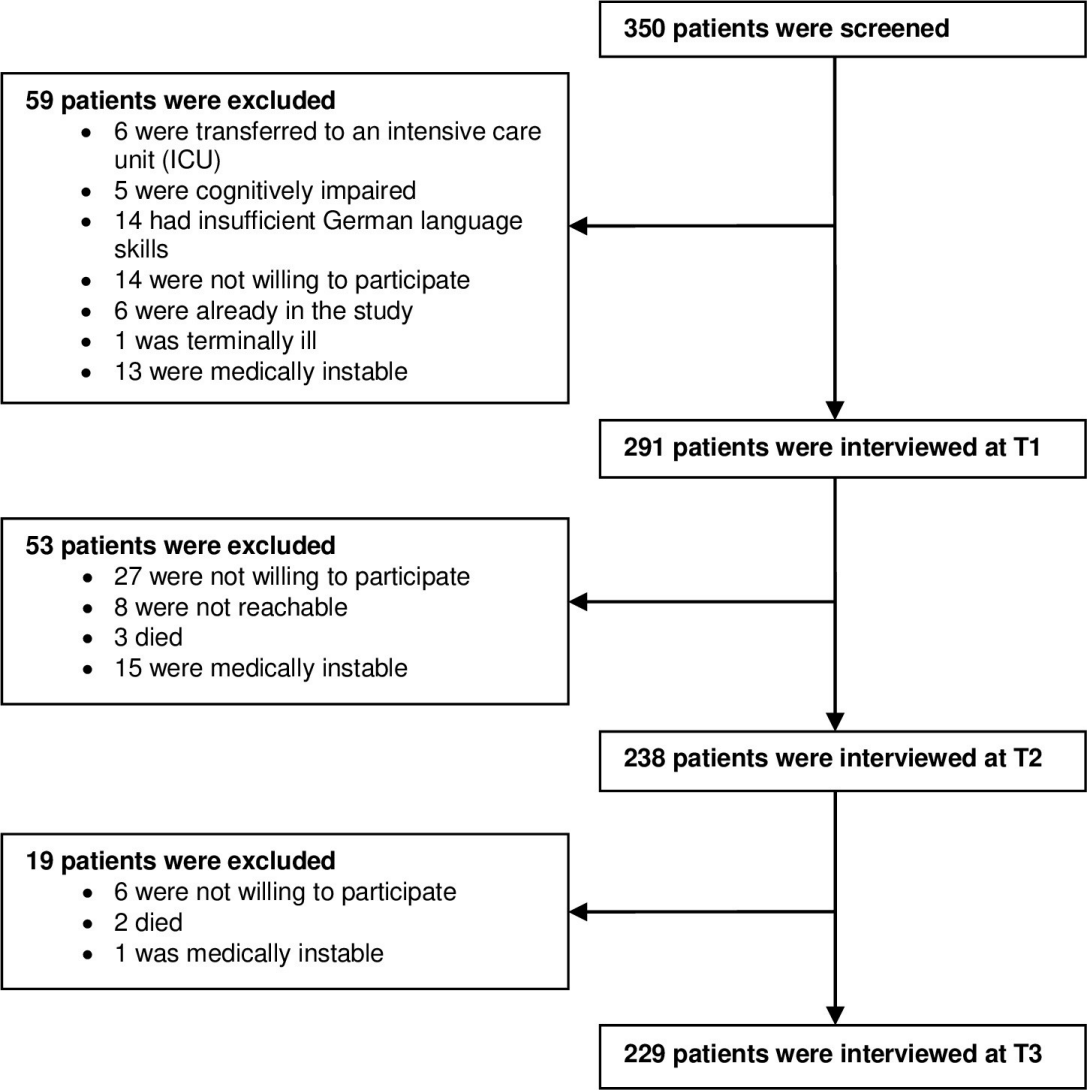
Flow chart of patient selection across the three time points: ED admission (T1), 7 days after (T2), and 30 days (T3).

Of the 350 screened patients, 291 (83%) agreed to be interviewed at baseline (T1, on average 10.5 hours after entering the ED). The patients who dropped out before T1 were significantly older (*p* < 0.01) and predominantly male (p < 0.01). Of the 291 patients, 238 (82%) were interviewed seven days following ED admission (T2). The patients who dropped out before T2 were significantly older (*p* < 0.05; no gender differences, *p* = 0.895). Of the 238 patients, 229 (96%) agreed to participate in the follow-up interview 30 days after ED admission (T3). The patients who dropped out before T3 were significantly older (*p* < 0.01; no gender differences, *p* = 0.053). Fig 1 presents a detailed description of the patient flow. The final sample consisted of 229 patients.

The descriptive statistics and internal consistency of the variables are presented in Table 1. Most of the patients were male and had a cardiac disease, and the patients were on average 67 years old.

**Table 1:** Descriptive statistics of the study variables.

N = 229	N (%)	Mean (SD)	Range
*Demographics*			
Gender			
Male	169 (74)		
Female	60 (26)		
Age	229	66.5 (12.8)	24–100
*Medical diagnosis*			
Lower respiratory tract infection	34 (15)		
Cardiac disease	193 (85)		
*Affective well-being*			
Negative affect at T1 (5–25; α = 0.69)	228	8.5 (3.4)	5–20
Negative affect at T2 (5–25; α = 0.70)	229	7.5 (3.1)	5–21
Negative affect at T3 (5–25; α = 0.64)	225	6.4 (2.1)	5–16
Positive affect at T1 (5–25; α = 0.56)	229	14.5 (3.4)	6–25
Positive affect at T2 (5–25; α = 0.72)	228	18.7 (3.5)	9–25
Positive affect at T3 (5–25; α = 0.66)	224	17.4 (3.7)	9–25
*Personal and social resources*			
Personality			
Emotional stability (1–10)	229	7.3 (1.7)	3–10
Trait resilience (11–77; α = 0.83)	225	63.2 (7.6)	41–77
Social support (5–35; α = 0.83)	223	31.0 (4.1)	16–35
*Health-related variables*			
Self-rated health (0–100)	225	58.8 (18.8)	10–100
Psychological comorbidity (6–30; α = 0.81)	229	11.6 (4.5)	6–23
Multimorbidity	229	3.7 (2.4)	1–12

T1–T3: ED admission (T1), 7 days after (T2), and 30 days after (T3); α: Cronbach’s alpha

Table 2 shows the intercorrelations of independent variables. The strongest correlation was between self-rated health and positive affect, followed by the correlation between morbidity and age.

**Table 2:** Pearson correlation matrix of independent variables.

	1.	2.	3.	4.	5.	6.	7.	8.	9.	10.
*Demographic characteristics*										
1. Gender	-									
2. Age	-.17*	-								
*Personal and social resources*										
3. Emotional stability	.06	.00	-							
4. Trait resilience	.04	.01	.33***	-						
5. Positive affect (T1)	.12	-.09	.21***	.26***	-					
6. Negative affect (T1)	-.09	-.27***	-.25***	-.21**	-.39***	-				
7. Social support	.00	-.20**	.11	.27***	-.24***	-.08	-			
*Health-related variables*										
8. Self-rated health	.18**	-.05	.07	.16*	.46***	.15*	.13	-		
9. Psychological comorbidity	-.14*	.00	-.30***	-.30****	-.26***	.37***	-.27***	-.24***	-	
10. Multimorbidity	-.27***	.42***	-.03	-.05	-.15*	-.08	-.22***	-.20**	.16*	-

* p < 0.05,

** p < 0.01,

*** *p* < 0.001

T1: ED admission

**Fig 2:**
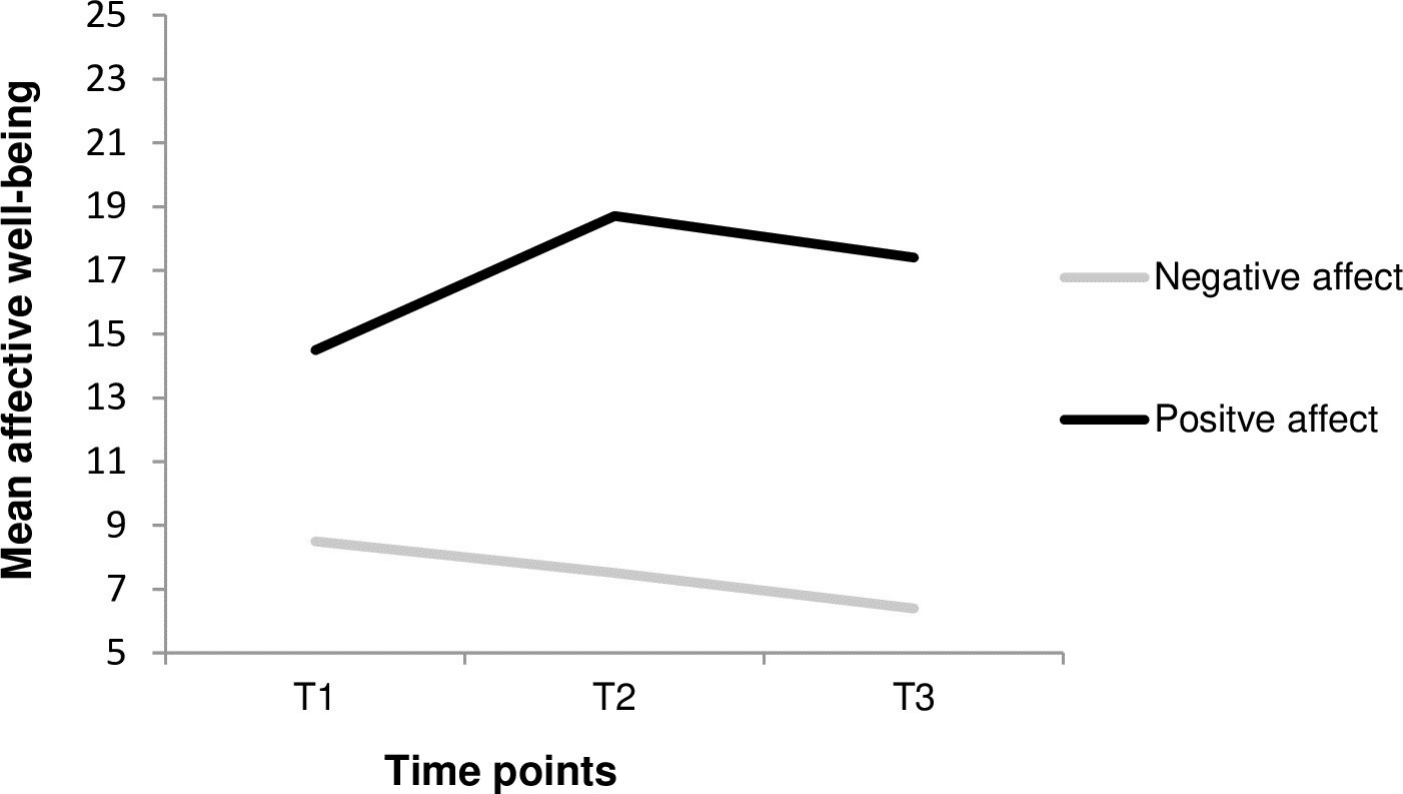
Course of negative and positive affect across the three time points (ED admission, (T1), 7 days (T2) and 30 days after (T3)).

Fig 2 illustrates the means of positive and negative affect across the three time points (T1–T3).

On average, negative affect decreased constantly across the time points, whereas positive affect increased from T1 to T2 and decreased from T2 to T3 (Table 1). Paired-sample *t*-tests showed significant differences between all the means for negative and positive affect. The mean negative affect was significantly higher at T1 than at T2, and the mean at T2 was higher than at T3 (all *p* < .001). In addition, the mean positive affect was significantly lower at T1 than at T2, whereas the mean at T2 was higher than at T3 (all *p* < .001). The negative and positive affect were negatively related at all three time points: r = -39 (T1), r = -.36 (T2), and r = -.31 (T3), all *p* < .001.

Regression analyses with negative affect at ED admission as the criterion showed that better personal resources, such as higher levels of positive affect, trait resilience, and emotional stability, were significantly associated with lower levels of negative affect (Table 3).

**Table 3:** Standardised regression coefficients indicating associations with patients’ affective state at ED (T1).

	Simple regression analysis		Multiple regression analysis	
	*Neg*. *affect*	*Pos*. *affect*	*Neg*. *affect*^*1*^	*Pos*. *affect*^*2*^
*Demographic characteristics*				
Gender (1 = male, 0 = female)	-.09	.12		-.01
Age	-.27***	-.09	-.29***	
*Personal resources*				
Emotional stability	-.25***	.21**	-.09	.08
Trait resilience	-.21**	.26***	-.03	.09
Positive affect (T1)	-.39***		-.34***	
Negative affect (T1)		-.39***		-.29***
*Social resources*				
Social support	-.08	.24***		.13*
*Health-related variables*				
Self-rated health	-.15*	.46***	.07	.37***
Psychological comorbidity	.37***	-.26***	.24**	.08
Multimorbidity	-.08	-.15*		.03

* p < 0.05,

** *p* < 0.01,

*** p < 0.001

ED: emergency department; T1: ED admission; LRTI: lower respiratory tract infection

^*1*^F(6,214) = 16.62, *p* < 0.001, R^2^ = .30

^*2*^F(8,212) = 13.53, *p* < 0.001, R^2^ = .34

Furthermore, more severe health-related conditions in addition to lower self-rated health and higher psychological comorbidity were significantly correlated with lower negative affect. Older age was significantly related to lower negative affect, and no correlation was found with negative affect for gender, social support, and multimorbidity. Regarding positive affect at ED admission, personal and social resources (i.e. higher emotional stability, higher trait resilience, stronger social support, and lower negative affect) were significantly associated with higher positive affect. Furthermore, all health-related variables (i.e. higher self-rated health, lower psychological comorbidity, and lower multimorbidity) were associated with higher positive affect. Demographic characteristics were not related to positive affect.

Results from multiple regression analyses revealed that older age, higher positive affect, and lower psychological comorbidity predicted lower negative affect at ED admission (Table 3). The strongest predictor was positive affect (β = -.34, *p* < 0.001), followed by age (β = -.29, *p* < 0.001), and psychological comorbidity (β = .24, p < 0.01). However, trait resilience, emotional stability, and self-rated health lost their predictive power. The multiple regression analyses also showed that lower negative affect, stronger social support, and higher self-rated health predicted higher positive affect at ED admission. The strongest predictor was self-rated health (β = 0.37, *p* < 0.001), followed by negative affect (β = -0.29, *p* < 0.001) and social support (β = 0.13, *p* < 0.05), whereas emotional stability, trait resilience, psychological comorbidity, and gender were not predictive.

Table 4 presents the predictors of affective well-being at 7 and 30 days after ED admission.

**Table 4:** Predictors of patients’ negative and positive affect 7 days (T2) and 30 days (T3) after ED admission controlled for baseline level (T1).

	Negative affect		Positive affect	
	*T2*^*1*^	*T3*^*2*^	*T2*^*3*^	*T3*^*4*^
*Baseline level (T1)*				
Negative affect	.21**	-.05		
Positive affect			.25***	.17*
*Demographic characteristics*				
Gender (1 = male, 0 = female)	-.12		.08	.10
Age	-.13*	-.16*		
*Personal resources*				
Emotional stability	-.22**	-.04	.07	.10
Trait resilience			.10	.11
Positive affect (T1)	.03			
Negative affect (T1)			-.06	.02
*Social resources*				
Social support			.14*	.08
*Health-related conditions*				
Self-rated health	-.02	-.07	.12*	.00
Psychological comorbidity	.24**	.27**	-.04	-.06
Multimorbidity			-.09	-.14*

* p < 0.05,

** *p* < 0.01,

*** *p* < 0.001

LRTI: lower respiratory tract infection; T1: ED admission; T2: day 7; T3: day 30

^*1*^F(7, 216) = 13.57, *p* < 0.001, R^2^ = .30

^*2*^F(5, 214) = 4.96, *p* < 0.001, R^2^ = .11

^*3*^F(9, 210) = 11.12, *p* < 0.001, R^2^ = .29

^*4*^F(9, 217) = 4.53, *p* < 0.001, R^2^ = .16

Regarding negative affect, we found that higher psychological comorbidity and younger age predicted higher negative affect at T2 and T3. Higher negative affect was also predicted by lower emotional stability, but only at T2. We found no predictive power for gender, self-rated health, or positive affect at T2 or for emotional stability or self-rated health at T3.

Multiple regression analyses further revealed that stronger social support and higher self-rated health predicted higher positive affect at T2. Additionally, lower positive affect at T3 was predicted by higher multimorbidity. Nonetheless, gender, emotional stability, trait resilience, negative affect, and psychological comorbidity lost their predictive power at T2 and T3. Furthermore, we found no significant effect for multimorbidity and self-rated health at T2 and T3, respectively.

In terms of baseline levels, a higher initial level at T1 was significantly related to a higher level of the corresponding affect at T2 (Table 4). A higher level of positive affect at baseline (T1) exhibited an even higher level at T3.

## Discussion

Only a few studies to date have investigated the course and determinants of affective well-being after a medical ED admission. In view of this existing research gap, our study makes two main contributions. First, it focuses on medical patients’ positive and negative affect instead of on their emotional distress (such as anxiety and depression). Second, our study considers personal and social resources as well as health-related variables as predictors of patients’ affective well-being.

The current study showed adaptation in terms of affective well-being in patients with cardiac diseases or lower respiratory tract infections following an ED admission by increasing positive affect and decreasing negative affect. Furthermore, we found a strong inverse correlation between positive and negative affect at ED admission, which decreased over time. This result is supported by the DMA model [11], which assumes an inverse relationship between positive and negative affect in times of stress compared to low-stress periods because of more simplified and rapid information processing. Thus, our findings suggest that ED patients’ positive and negative affective systems become more independent over time. This experience of greater co-occurrence of positive and negative affect may help patients to enhance their well-being.

Our results are in line with the DMA model, according to which the timing of the affect matters [9], that is, elevation in positive affect during times of heightened stress is particularly important in the regulation of negative affective states. Accordingly, our findings imply that the presence of positive emotions is important for the preservation of patients’ well-being at ED admission and that a deficit in positive emotions increases vulnerability to negative emotions.

A further aim of this study was to examine the role of personal and social resources for patients’ affective well-being. Our findings confirm that a large range of these resources is associated with patients’ affective well-being. Regarding personal resources, emotional stability and trait resilience were related to negative affect at all three time points, a result supported by the existing literature [10,12]. These effects of personal resources also held for positive affect. However, only the relationship between emotional stability at T2 and negative affect remained significant when controlling for demographic characteristics, baseline affect levels, health-related variables, and social resources. This suggests that higher emotional stability helps patients sustain lower negative affect especially during hospitalisation, when stress experience is still high because of medical instability or slow recovery. Regarding social resources, namely social support, we found no significant relations with negative affect, a finding inconsistent with our expectations. An explanation may be that we concentrated on affect states instead of emotional distress, which was used in most of the previous studies [7,16]. However, we found that stronger social support was associated with higher positive affect over time and, further, identified it as a predictor of higher positive affect at ED and seven days later, when controlling for confounders. Evidence of this beneficial effect of social resources can also be found elsewhere in the literature. Manne and colleagues [32] showed that after active treatments, supportive responses from family and friends of cancer patients were associated with a faster recovery of positive affect over time. Thus, we suggest that social support may be a protective factor in medical patients after an ED admission and subsequent hospitalisation.

With regard to health-related variables, our findings confirm that psychological comorbidity is associated with higher negative affect at ED admission as well as at 7 and 30 days later. This result supports findings in the existing literature [2,18,33] suggesting that psychological comorbidity is a predisposing factor for higher levels of negative affect in medical patients throughout the period from ED admission to 30 days later. Our results further confirm that lower self-rated health is associated not only with higher negative affect, but also with lower positive affect. However, this effect remained significant only for positive affect at ED and T2, when controlling for confounders. An explanation could be that physical and affective well-being are not necessarily related especially in older age, as research on ageing has shown (paradox of well-being) [34]. Thus, our findings suggest that patients’ perception of a poor health state is a risk factor for lower levels of positive affect in medical patients in the period from ED admission to hospitalisation. We also assume that, like self-rated health, multimorbidity (i.e. more active medical diagnoses) is associated with lower positive affect. However, multimorbidity was only predicted for positive affect at T3. This suggests that the medical condition becomes more important for positive affect after discharge from hospital.

### Limitations

Our study sample included significantly more males than females. This could be explained by the fact that cardiovascular illnesses (especially myocardial infarction) occur more frequently in males [35]. Considering also the fact that dropouts were associated with older age, the age effects found in this study should be interpreted carefully (possible selection effect). Furthermore, the results were not controlled for different acute medical conditions at ED admission. Hence, the generalisability of the study is somehow limited for two reasons: (1) only patients with lower respiratory tract infections and cardiac diseases were considered, and (2) data were gathered from a single hospital. A broader set of medical diagnoses as well as a multicentre design could increase the generalisability of future studies. Another limitation involves the regression models, which explained a moderate amount of variance at T3. Thus, some variability in negative and positive affect has to be accounted for by other variables, such as social factors (e.g., resumption of work, leisure behaviour) or clinical characteristics (e.g., activity of daily living, medical stability). Finally, multilevel modelling or latent growth modelling would be more suitable for repeated measurements. Initially, we computed growth models using Mplus. However, the goodness of fit indices were not acceptable for negative affect (x^2^(8) = 5.52, p = 0.019; CFI = 0.93; TLI = 0.78; RMSEA = 0.142, 90% CI = 0.046–0.266) and positive affect (x^2^(8) = 174.80, p < 0.001; CFI = 0.00; TLI = -4.96; RMSEA = 0.883, 90% CI = 0.775–0.995).

### Clinical implications

Overall, our study highlights the importance of personal and social resources (emotional stability and social support) and of health-related variables (psychological comorbidity, self-rated health, and multimorbidity) for affective well-being over time. The results suggest that early identification of a risk of psychological comorbidity, and of poor self-rated health, using routine screenings could facilitate the provision of appropriate interventions or treatments. Furthermore, ED and ward staff could be trained in health-promoting programs to foster patients’ emotional stability and social support. Considering the long waiting hours and limited patient care at ED and during hospitalisation, our study suggests strongly that investing more time and effort in patients could significantly improve their affective well-being. Our study also revealed the detrimental effect of multimorbidity on positive affect one month following ED admission. Thus, patients’ medical condition may be more important than their personal and social resources.

### Conclusions

This study offers innovative insights into adaptation of affective well-being in medical ED patients by showing that emotional recovery is associated with an increase in the co-occurrence of positive and negative affect. Health-related conditions as well as personal and social resources are crucial for emotional adaptation after ED admission. Future research should identify thresholds in patients’ affect levels to detect potentially at-risk populations. In addition, further studies should clarify whether better affective adaptation leads to better health outcomes, such as lower readmission rates, length of hospital stay, and better quality of life. Finally, future research should investigate whether well-adapted medical ED patients have beneficial effects on the resources of medical staff within the hospital setting.

## Supporting information

S1 Dataset Dataset of the study. (XLS)
